# Accurate estimation of intravoxel incoherent motion parameters based on implicit neural representation

**DOI:** 10.1002/mp.70599

**Published:** 2026-07-28

**Authors:** Yunxiang Li, Yen‐Peng Liao, Yan Dai, Jie Deng, You Zhang

**Affiliations:** ^1^ Department of Radiation Oncology UT Southwestern Medical Center Dallas Texas USA

**Keywords:** diffusion‐weighted imaging, implicit neural representation, intravoxel incoherent motion

## Abstract

**Background:**

Intravoxel incoherent motion (IVIM) diffusion‐weighted imaging has important value in treatment response monitoring. However, traditional voxel‐wise independent fitting methods are highly sensitive to noise and do not utilize spatial correlation, resulting in unstable parameter estimation. Although existing deep learning methods have shown improvements, they are still limited by local receptive fields.

**Purpose:**

To address this, we propose a two‐stage IVIM parameter estimation framework based on Implicit Neural Representation (IVIM‐INR).

**Methods:**

Our IVIM‐INR method achieves global spatial perception through coordinate encoding and enhances spatial context modeling by leveraging local 3D patch information from multi‐*b*‐value images. The first stage INR performs signal denoising, and the second stage INR accurately fits IVIM parameters.

**Results:**

Evaluation on brain digital phantoms, AAPM breast IVIM‐dMRI Challenge data, and clinical Glioblastoma (GBM) patient data demonstrates significant advantages of the proposed method over existing techniques. In brain simulation data, when SNR = 50, the normalized mean absolute errors (NMAEs) in tumor regions were 0.16±0.10 for $D_{p}$, 0.02±0.01 for $D_{t}$, and 0.07±0.05 for $F_{p}$, all lower than comparison methods. In the tumor tissues of the 100 cases from the AAPM breast IVIM‐dMRI challenge dataset, $F_{p}$ error was reduced by 58% compared to ConvNet. Intraclass correlation coefficient (ICC) analysis of real clinical data indicates that our method achieves the best performance with an ICC of $D_{t}$ in normal tissues reaching 0.629.

**Conclusions:**

By combining INR's continuous function modeling capability with spatial‐aware feature design, IVIM‐INR overcomes the inherent limitations of traditional methods under noisy conditions, providing a more reliable tool for clinical IVIM quantitative analysis.

## 1 INTRODUCTION

Intravoxel incoherent motion (IVIM) diffusion‐weighted imaging, based on a biexponential model, can simultaneously quantify tissue diffusion and microcirculation perfusion characteristics, providing unique advantages for functional imaging without contrast agents.^1^ The IVIM model decomposes the diffusion‐weighted signals into true diffusion and pseudo‐diffusion components, where pseudo‐diffusion reflects the random motion of blood in the capillary network.^2^ This dual information makes IVIM valuable in clinical applications such as tumor diagnosis and grading,^3^, ^4^ early assessment of treatment response,^5^ and detection of ischemic diseases.^6^ Particularly in patient populations where contrast agent use is restricted, IVIM provides an alternative approach for assessing tissue perfusion.^7^ Several signal models have been proposed for IVIM analysis beyond the classical biexponential formulation. The tri‐exponential model introduces a third compartment to better capture complex diffusion behaviors in highly vascularized organs such as the liver.^8^ Diffusion kurtosis imaging extends the signal model by incorporating a kurtosis term to quantify non‐Gaussian water diffusion.^9^ Model‐free approaches, such as the area under the signal decay curve (AUC), provide non‐parametric alternatives that avoid assumptions about the number of exponential components.^10^ Among these, the biexponential model remains the most widely used and validated framework for IVIM analysis, with parameters (tissue diffusion and microcirculation perfusion) that carry clear physiological meaning. It has been extensively validated across diverse clinical applications. This study therefore adopts the biexponential model as the foundation, while the framework's extensibility to alternative models is discussed in Section 4.

However, accurate estimation of IVIM parameters faces significant technical challenges. The nonlinear nature of the biexponential model makes parameter fitting extremely sensitive to noise, especially under low signal‐to‐noise ratio (SNR) conditions common in clinical practice. Traditional voxel‐wise fitting methods, such as least squares, ignore spatial correlation between neighboring voxels, resulting in parameter maps with severe noise contamination and discontinuities.^11^ This instability is particularly pronounced in the estimation of perfusion fraction ($F_{p}$) and pseudo‐diffusion coefficient ($D_{p}$), as these parameters are highly sensitive to the rapidly decaying signal component.^12^ In clinical practice, this unreliable parameter estimation limits the quantitative application of IVIM, affecting diagnostic accuracy and complicating treatment decision‐making. To improve the stability of parameter estimation, researchers have explored various optimization algorithms and regularization techniques. The Trust‐Region Reflective (TRR)^13^ algorithm, as a constrained optimization method, handles parameter boundary constraints through iterative searching within a trust region, improving fitting robustness to some extent. However, this algorithm still relies on voxel‐wise processing and fails to utilize spatial correlation information. The emergence of deep learning has brought new possibilities for IVIM parameter estimation.^14^, ^15^ IVIMNet, as one of the first specifically designed deep learning methods, learns the mapping from diffusion‐weighted signals to IVIM parameters through neural networks.^15^ However, IVIMNet still employs a voxel‐wise processing strategy and fails to fully utilize spatial context information. Later proposed convolutional neural network (ConvNet)^14^ methods introduced spatial information processing capabilities, aggregating local neighborhood features through convolution operations. However, inherent limitations of convolutional networks also apply to the IVIM estimation: the local receptive field limits perception of global structures, the translation invariance can be problematic for medical images where anatomy is structured, and the position/location carries diagnostic meaning.^16^ For ConvNet, there is also a lack of flexibility in handling spatial correlations at different scales.

Implicit neural representation (INR), as an emerging continuous function modeling paradigm, provides a new perspective for medical image parameter estimation.^17^ Unlike discrete voxel representations, INR parameterizes images as continuous mappings from coordinates to values, learning this mapping relationship through neural networks. This representation offers multiple advantages: first, coordinate input grants the network natural global perception ability to model long‐range spatial dependencies;^18^ second, continuous function representation avoids discretization errors; finally, INR's implicit regularization properties help produce smooth, physically plausible parameter distributions. Recent studies have successfully applied INR to tasks such as MRI reconstruction^19^, ^20^ and CT metal artifact reduction,^21^ demonstrating its potential in medical imaging. The choice of activation function plays a crucial role in INR's expressive capability. ReLU activation functions tend to learn piecewise linear functions and can effectively suppress high‐frequency noise,^22^ making them ideal for the first stage, where we fit DWI images at different *b*‐values for denoising. Notably, while INR with ReLU activation without additional frequency encoding may lose high‐frequency information,^23^ we effectively preserve high‐frequency structural details by incorporating local multi‐*b*‐value patches as input, since the structure information across different *b*‐value images is highly correlated. The second stage, however, requires fitting IVIM model parameters, which is a more complex task due to the biexponential nature of the IVIM model with multiple parameters. Periodic activation functions like Sinusoidal Representation Network (SIREN)^24^ can better capture complex parameter relationships and spatial variations, making them suitable for this nonlinear parameter fitting task. Based on this consideration, this study proposes IVIM‐INR, a two‐stage IVIM parameter estimation framework based on implicit neural representation. The first stage employs ReLU‐activated INR for multi‐*b*‐value DWI signal denoising, utilizing spatial continuity constraints and multi‐*b*‐value mapping to suppress random noise. The second stage uses SIREN‐activated INR to accurately fit IVIM parameters from denoised signals, capturing detailed features of microstructure. Our main contributions include: (1) To our knowledge, this is the first introduction of implicit neural representation to IVIM parameter estimation, proposing a new method based on continuous function modeling; (2) Design of a two‐stage framework that achieves a balance between denoising and detail preservation through complementary activation function properties; (3) Comprehensive validation on multiple datasets, including brain simulations at different noise levels, AAPM breast IVIM‐dMRI Challenge data, and real clinical data, demonstrating superior performance and clinical application potential of the method.

## 2 METHODS

### 2.1 Problem formulation

The IVIM model describes the decay of diffusion‐weighted signals through a biexponential function:

(1) $$\frac{I(b)}{I_{0}}=F_{p}\cdot \exp\left(-b\cdot D_{p}\right)+\left(1-F_{p}\right)\cdot \exp\left(-b\cdot D_{t}\right)$$

where I(b) represents the diffusion‐weighted image intensity at diffusion sensitivity factor b, and $I_{0}=I\left(0\right)$ is the unweighted reference intensity. Model parameters include: $D_{t}$ (tissue diffusion coefficient, reflecting true water molecule diffusion), $D_{p}$ (pseudo‐diffusion coefficient, reflecting microcirculation perfusion), $F_{p}$ (perfusion fraction, representing the proportion of perfusion component).

Traditional voxel‐wise fitting treats each spatial location as an independent optimization problem, ignoring the inherent spatial continuity of medical images. We reformulate IVIM parameter estimation as learning a continuous parameter field $\Theta :R^{3}\to R^{3}$, where $\Theta \left(v\right)=[D_{p}\left(v\right),D_{t}\left(v\right),F_{p}\left(v\right)]$ represents IVIM parameters at spatial location v. This continuous representation naturally encodes spatial regularization, helping produce smooth, anatomically reasonable parameter distributions. Importantly, our approach employs a self‐supervised learning strategy without requiring “ground‐truth” IVIM parameter values, which are virtually impossible to obtain in real clinical practice.

### 2.2 Overview of the two‐stage IVIM‐INR framework

**FIGURE 1:**
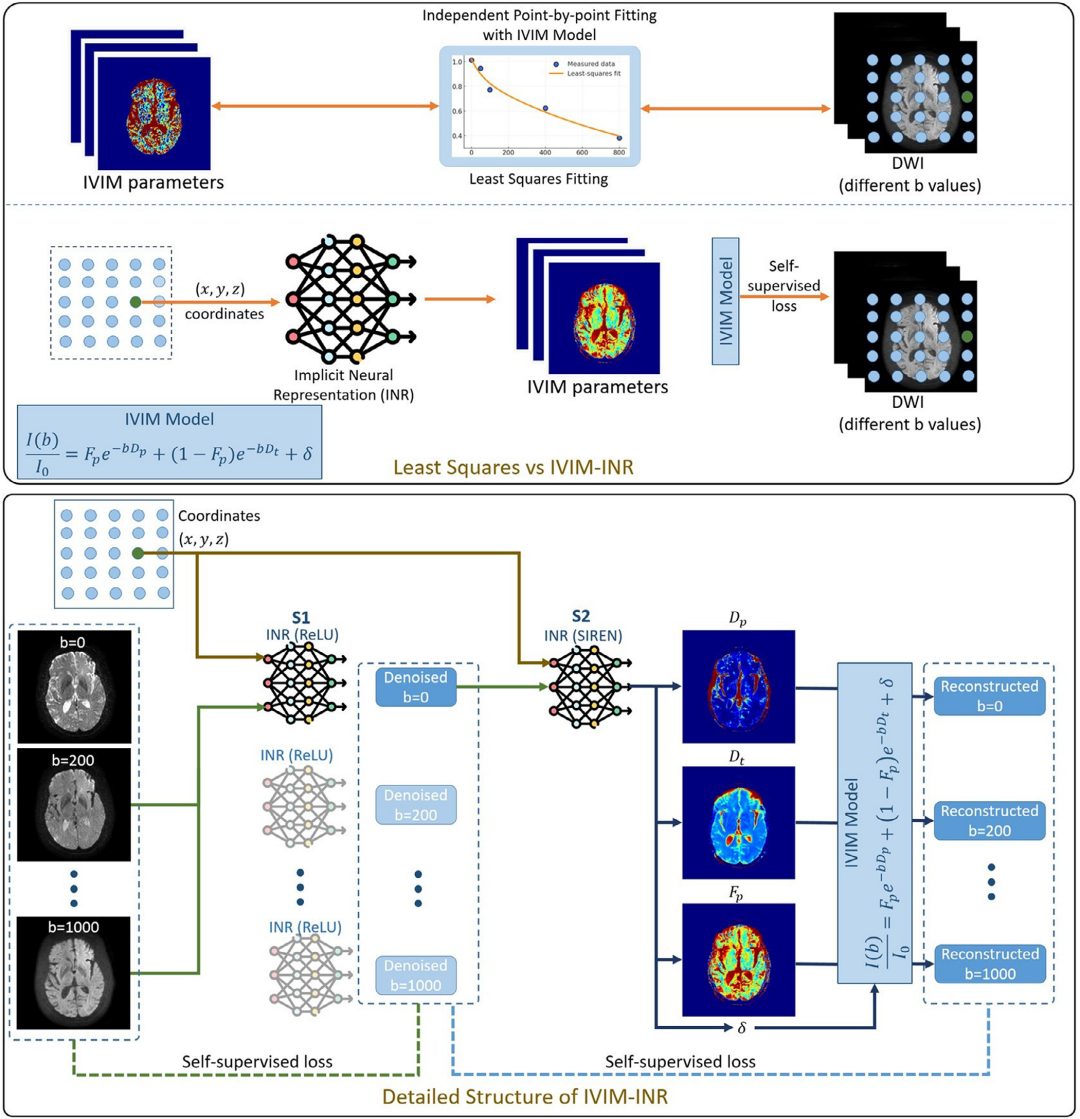
The IVIM parameter estimation framework based on implicit neural representation. The upper part contrasts traditional voxel‐wise methods (least squares) with IVIM‐INR: traditional methods process each voxel independently, while IVIM‐INR achieves global perception through coordinate encoding. The lower part details the two‐stage architecture. In the first stage (S1), a separate ReLU‐activated INR is independently trained for denoising each *b*‐value; when denoising a given target *b*‐value, the inputs to its dedicated INR are the spatial coordinates (x,y,z) and the 3D patches from all *other* *b*‐values (excluding the target *b*‐value itself). The semi‐transparent INR blocks indicate conceptually identical but independently trained INRs for the remaining target *b*‐values. The second stage (S2) employs a SIREN‐activated INR that takes coordinates and the denoised b=0 patches as input, outputting the IVIM parameters ($D_{p}$, $D_{t}$, $F_{p}$). These parameters are fed into the biexponential IVIM model to reconstruct signals at all *b*‐values, governed by a self‐supervised loss against the denoised signals from Stage 1. The two‐stage design effectively balances noise suppression and detail preservation.

**FIGURE 2:**
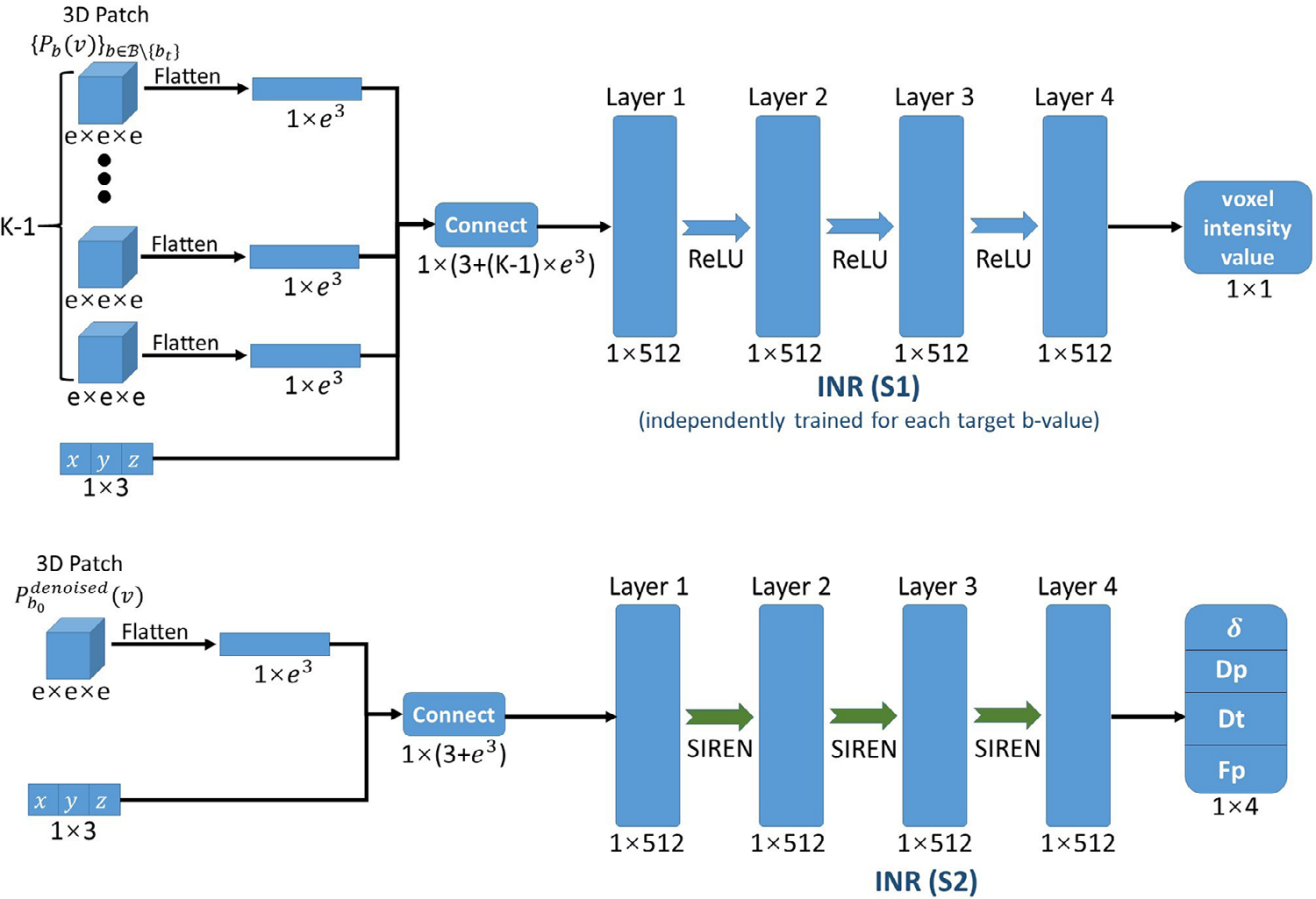
Detailed network architecture of the two‐stage IVIM‐INR framework. Stage 1 (S1, top) is a 4‐layer MLP with ReLU activations. It takes the concatenation of spatial coordinates (x,y,z) and flattened 3D patches (of size e×e×e) from the K−1 non‐target *b*‐values (i.e., $B\setminus \{b_{t}\}$ for target *b*‐value $b_{t}$) as input, and outputs the denoised voxel intensity at the target *b*‐value $b_{t}$. An independent S1 network is trained for each target b‐value. Stage 2 (S2, bottom) is a 4‐layer MLP with SIREN activations. It takes the concatenation of spatial coordinates and the flattened 3D patch of the denoised b=0 image as input, and outputs the IVIM parameters ($D_{p}$, $D_{t}$, $F_{p}$); a voxel‐wise offset δ is additionally learned and added to the IVIM model as described in Equation (6). The predicted parameters are fed into the biexponential IVIM model to reconstruct signals at all b‐values, which are compared against the denoised target signals for self‐supervised learning. All hidden layers contain 512 units.

Figure 1 illustrates the high‐level architecture of the proposed IVIM‐INR framework, and Figure 2 provides a detailed layer‐by‐layer view. Unlike traditional methods that fit voxels independently, we model the entire parameter field using implicit neural representations, achieving global spatial perception through coordinate input. The framework consists of two complementary stages:

The first stage (S1) employs a multi‐layer perceptron (MLP) with ReLU activation for signal denoising. ReLU's piecewise linear property effectively suppresses high‐frequency noise while maintaining the main signal structure. A separate denoising network is trained for each *b*‐value, with input consisting of spatial coordinates and 3D patches from other *b*‐values, achieving the sharing and communication of multi‐*b*‐value information. The use of multi‐*b*‐value inputs also enables the first denoising stage to preserve meaningful high‐frequency structural details that are shared across the b‐value images (as compared to noise distributions, which are more random), since the non‐frequency‐encoded INR of the first stage can fail to capture high‐frequency structural details if used alone. Specifically, the S1 network consists of an input layer, multiple hidden layers with ReLU activation, and a linear output layer that produces the denoised signal value for the target *b*‐value. Weights are initialized using Xavier uniform initialization to facilitate stable training.

The second stage (S2) uses INR with Sinusoidal Representation Network (SIREN) activation to accurately fit IVIM parameters from denoised signals. SIREN uses periodic sinusoidal activation functions together with a specialized weight initialization scheme designed to keep signal propagation well‐conditioned across layers, following Sitzmann et al.^24^ This periodic activation can capture high‐frequency details of parameter distributions, particularly important for tissue boundaries and heterogeneous regions. This stage takes coordinates and 3D patches of the denoised b=0 image as input. The SIREN backbone extracts high‐dimensional features, which are then mapped to IVIM parameters through a dedicated parameter head: a linear layer projects the feature vector to four outputs corresponding to $D_{p}$, $D_{t}$, $F_{p}$, and offset δ. Physical constraints are enforced through a sigmoid output activation followed by linear scaling, bounding each output to its physiologically plausible range (the specific bounds are given in the Implementation Details below).

### 2.3 Spatial‐aware feature design

To fully utilize spatial information for image denoising, we designed a feature representation strategy combining global coordinates and local patches. Let B denote the full set of K b‐values acquired in the imaging protocol, and let $b_{0}\in B$ specifically denote the unweighted reference image (with $b_{0}=0$ by convention, analogous to $I_{0}$). For each b∈B, we extract a 3D patch $P_{b}\left(v\right)\in R^{e^{3}}$ of size e×e×e centered at a voxel v. Here, a 3D patch refers to a cubic neighborhood region extending in all three spatial dimensions around the target voxel. For example, when e=3, the patch contains the target voxel and its 26 nearest neighbors, yielding 27 voxel values that encode local spatial context to help the network distinguish signal structure from random noise.

For first‐stage denoising, an independent INR $f_{S1}^{\left(b_{t}\right)}$ is trained for each target b‐value $b_{t}\in B$ (here the subscript t denotes the target *b*‐value currently being denoised). The INR takes the spatial coordinates together with 3D patches drawn from all *other* *b*‐values as input, and outputs the denoised intensity at $b_{t}$:

(2)

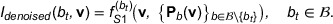

The target b‐value's own (noisy) image is intentionally excluded from the input so that the network cannot trivially copy noisy values from the target patch; instead, it must infer the clean signal at $b_{t}$ from the spatial structure encoded by the other *b*‐values' patches together with the global coordinate v. This design enables the network to leverage the high correlation of signals and low correlation of noise across multiple b‐values, improving the efficacy of denoising. Coordinates v provide global position information, allowing the network to utilize spatial signal continuity for denoising.

For second‐stage parameter fitting, the INR $f_{S2}$ maps coordinates and the denoised b=0 patch directly to the IVIM parameters and the offset:

(3) $$\left[D_{p}\left(v\right),\;D_{t}\left(v\right),\;F_{p}\left(v\right),\;\delta \left(v\right)\right]=f_{S2}\;\left(v,\;P_{b_{0}}^{denoised}\left(v\right)\right),$$

where $P_{b_{0}}^{denoised}$ is the denoised b=0 image patch produced by its dedicated S1 network (i.e., $f_{S1}^{\left(b_{0}\right)}$). The high‐quality anatomical information after denoising provides a stable spatial context for parameter estimation. The choice of patch size e is detailed in Section 3.5. Experiments show that e=3 provides sufficient spatial context while avoiding excessive smoothing.

### 2.4 Optimization objectives and constraints

The two stages of the IVIM‐INR network are separately and sequentially trained (stage 1 first, followed by stage 2) via a “one‐shot” learning scheme using mean squared error losses.

For the first stage, each S1 network $f_{S1}^{\left(b_{t}\right)}$ is trained independently by minimizing a per‐target‐b‐value MSE loss between its denoised prediction and the original noisy intensity at $b_{t}$:

(4) $$L_{S1}^{\left(b_{t}\right)}=\frac{1}{N}\sum_{j=1}^{N}{\left\|I_{denoised}\left(b_{t},v_{j}\right)-I_{noisy}\left(b_{t},v_{j}\right)\right\|}^{2},\;b_{t}\in B,$$

where N is the number of sampled voxels and $I_{noisy}\left(b_{t},v_{j}\right)$ is the original noisy intensity at b‐value $b_{t}$ and voxel $v_{j}$. The K S1 networks (one per b‐value in B) share no parameters and are trained in parallel, so this is equivalent to summing $L_{S1}^{\left(b_{t}\right)}$ over $b_{t}\in B$.

For the second stage, the IVIM parameters predicted by the single S2 network are substituted into the biexponential model (Equation 6) to reconstruct the intensity at every b‐value, and the reconstructions are compared against the denoised intensities from the first stage across all b‐values in B:

(5) $$L_{S2}=\frac{1}{N\;|B|}\sum_{j=1}^{N}\sum_{b\in B}{\left\|I_{pred}\left(b,v_{j}\right)-I_{denoised}\left(b,v_{j}\right)\right\|}^{2},$$

where $I_{pred}\left(b,v_{j}\right)$ is the IVIM‐model‐reconstructed intensity computed from the parameters $\left(D_{p},D_{t},F_{p},\delta \right)$ predicted by S2 at $v_{j}$ via Equation (6), and $I_{denoised}\left(b,v_{j}\right)$ is the denoised intensity output by the corresponding S1 network as defined in Equation (2).

Traditional IVIM fitting typically assumes that the normalized signal at *b* = 0 strictly equals 1, forcing the fitting curve through point (0,1). However, actual measured signals are affected not only by noise but also by inherent biases from hardware imperfections such as gradient nonlinearity, eddy currents, and residual gradients, even at nominally *b* = 0 (no diffusion gradient) conditions^25^. This hard constraint may lead to parameter estimation bias. As an alternative to fitting $I_{0}$ as a free parameter,^26^, ^27^ which similarly relaxes this hard constraint, we introduce an additive offset parameter δ in the normalized IVIM signal model to accommodate such systematic deviations:

(6) $$\frac{I(b)}{I_{0}}=F_{p}\cdot \exp\left(-b\cdot D_{p}\right)+\left(1-F_{p}\right)\cdot \exp\left(-b\cdot D_{t}\right)+\delta$$

The offset δ is applied to the entire fitting curve for each pixel, allowing the model to flexibly adapt to noise while maintaining the diffusion principle. To prevent excessive offset, we apply a mean‐squared regularization constraint on δ, encouraging it to approach zero. This strategy is uniformly applied across all evaluated methods to ensure fair comparison.

### 2.5 Implementation details

All methods are implemented in Python. The comparison methods (see Section 2.7 for details), IVIMNet and ConvNet, are also implemented using the PyTorch deep learning framework as IVIM‐INR, with hyperparameters configured according to their respective original publications. Traditional least squares and TRR fitting are implemented using the SciPy optimization library. During the preprocessing stage for all datasets, we employ Rician bias correction based on background noise,^28^ by calculating the mean of background noise and applying the correction formula $\sigma =\mu_{bg}/\sqrt{\pi /2}$, $I_{corrected}=\sqrt{\max(I_{measured}^{2}-2\sigma^{2},0)}$.^28^ This standardization method effectively reduces signal bias caused by Rician noise, particularly in low‐signal regions at high b‐values.

To ensure fair comparison, all methods employ identical parameter boundary constraints during fitting:^29^ $D_{p}\in \left[0.001,0.02\right]$ ${\mathrm{mm}}^{2}$/s, $D_{t}\in \left[0.00001,0.003\right]$ ${\mathrm{mm}}^{2}$/s, and $F_{p}\in \left[0.0,0.5\right]$. The offset parameter δ is additionally constrained to [−0.1,0.1]. These bounds encompass the physiologically plausible range of tissue IVIM parameters while preventing unrealistic values during optimization.

For network architecture, both stages of our network employ a 4‐layer fully connected structure with 512 hidden units per layer. The second‐stage network output layer applies a sigmoid activation, and each parameter is then linearly scaled to its predefined range ($D_{p}$, $D_{t}$, $F_{p}$) to enforce the above bounds. Training employs the Adam optimizer combined with cosine annealing learning rate scheduling. Initial learning rates are $5\times 10^{-4}$ for the first stage and $5\times 10^{-5}$ for the second stage. All experiments are conducted on an NVIDIA RTX 4090 GPU.

All deep learning‐based methods (IVIM‐INR, IVIMNet, and ConvNet) are trained using self‐supervised learning, where each case has a dedicated model trained independently on its own multi‐b‐value DWI data without requiring external training datasets. All deep learning‐based methods were optimized for 1000 epochs to ensure convergence. The source code of IVIM‐INR is available at https://github.com/Kent0n‐Li/IVIM‐INR.

### 2.6 Datasets

To comprehensively evaluate the performance of IVIM‐INR, we employed three complementary datasets:

#### 2.6.1 Brain digital phantom

Anatomically realistic IVIM simulation data constructed based on the Harvard‐Oxford brain atlas are used. Referring to the existing literature,^29^, ^30^, ^31^, ^32^, ^33^ we use the following tissue‐specific parameter values for: gray matter ($D_{t}=0.85\times 10^{-3}\;{\mathrm{mm}}^{2}/s$, $D_{p}=4.2\times 10^{-3}\;{\mathrm{mm}}^{2}/s$, $F_{p}=0.11$), white matter ($D_{t}=0.75\times 10^{-3}\;{\mathrm{mm}}^{2}/s$, $D_{p}=3.6\times 10^{-3}\;{\mathrm{mm}}^{2}/s$, $F_{p}=0.07$), and cerebrospinal fluid ($D_{t}=3.0\times 10^{-3}\;{\mathrm{mm}}^{2}/s$, $D_{p}=0$, $F_{p}=0$). To further assess robustness on pathological tissues, we instantiate three representative configurations of a tumor,^30^ which is placed in the left brain lobe: (1) $D_{t}=0.71\times 10^{-3}\;{\mathrm{mm}}^{2}/s$, $D_{p}=14\times 10^{-3}\;{\mathrm{mm}}^{2}/s$, $F_{p}=0.1$; (2) $D_{t}=1.14\times 10^{-3}\;{\mathrm{mm}}^{2}/s$, $D_{p}=9.79\times 10^{-3}\;{\mathrm{mm}}^{2}/s$, $F_{p}=0.13$; and (3) $D_{t}=1.01\times 10^{-3}\;{\mathrm{mm}}^{2}/s$, $D_{p}=19\times 10^{-3}\;{\mathrm{mm}}^{2}/s$, $F_{p}=0.18$.

Images for all b‐values are then generated based on these parameters, resulting in DWI images with 8 b‐values (0, 50, 100, 200, 400, 600, 800, and 1000 s/${\mathrm{mm}}^{2}$). To make the data more realistic, we apply multiplicative spatial modulations to the DWI images: a slow, smooth field with about 10% peak‐to‐peak variation to simulate spatial inhomogeneity such as that arising from B1 field non‐uniformity. Injecting ∼10%‐scale long‐range‐correlated spatial variation to emulate realistic imaging conditions is consistent with existing IVIM simulation practice; the AAPM IVIM‐dMRI challenge,^34^ for example, allows IVIM parameter values to vary within ±10% of nominal values with long‐range spatial correlation to emulate tissue heterogeneity. We then add noise to all b‐value images. We define the SNR using the mean b=0 magnitude within the brain region as the signal intensity. For each SNR level, we calculate the corresponding noise standard deviation based on the measured signal intensity. We then separately add simulated zero‐mean Gaussian noise with the calculated standard deviation to the real and imaginary channels of the original image, generating new magnitude images at the desired SNR levels; this procedure yields Rician‐distributed noise, consistent with the standard noise model in magnitude MRI.^28^ Note that these simulation settings, including the spatial modulations and noise, are applied identically to all methods evaluated, ensuring a fair comparison. Correspondingly, we simulate b=0 images with different SNR levels of 30, 40, 50, and 60. For DWI images with higher *b*‐values, the SNRs will be relatively lower due to decreased signal intensities.

#### 2.6.2 AAPM IVIM‐dMRI challenge data

The first 100 digital breast model cases from the AAPM IVIM‐dMRI challenge^34^ are used. This challenge utilizes the VICTRE (Virtual Imaging Clinical Trial for Regulatory Evaluation) Breast Model^35^ to provide realistic anatomical breast structures, and constructs IVIM parameters for different typical breast tissue types. The challenge generates DWI data based on these assigned IVIM parameters, creating a standardized testing platform with known “ground‐truth” values. It enables a comprehensive evaluation of method performance across different tissue environments with realistic tissue heterogeneity. Following the data usage guidelines,^34^ we excluded fat tissue from evaluation as it is normally suppressed in DWI imaging. We utilize 7 *b*‐values (0, 50, 100, 200, 500, 800, and 1000 s/${\mathrm{mm}}^{2}$) for IVIM fitting, and employ the 100 cases for self‐supervised learning evaluation, with all models trained on their respective data, requiring no train‐test split.

#### 2.6.3 Real clinical data

MR‐Linac multi‐timepoint DWI data collected from 7 glioblastoma (GBM) patients are used. Each scan contains 7 *b*‐values (0, 50, 100, 200, 500, 800, and 1000 s/${\mathrm{mm}}^{2}$) acquired during daily adaptive radiotherapy via a 1.5 T MR‐Linac. During preprocessing, we use FLAIR images from the same scan as reference, employing mutual information‐based deformable registration algorithms to correct DWI geometric distortions.^36^ This anatomical image‐guided distortion correction method is widely used in DWI preprocessing and can effectively reduce DWI deformations.^37^ All models are independently trained and tested on individual cases, following a self‐supervised learning paradigm without external training sets.

### 2.7 Comparison methods and evaluation metrics

Four representative methods were evaluated for comparison: traditional least squares,^38^ TRR,^13^ a deep learning voxel‐wise method (IVIMNet),^15^ and a convolutional neural network‐based method (ConvNet).^14^

Simulated data uses normalized mean absolute error (NMAE) to evaluate parameter estimation accuracy:

(7) $$\text{NMAE}=\frac{1}{N}\sum_{j=1}^{N}\frac{|\theta_{j}^{pred}-\theta_{j}^{true}|}{|\theta_{j}^{true}|}$$

where N is the number of voxels, j is the voxel index, and θ represents the parameter value.

Real data employs the intraclass correlation coefficient (ICC) to evaluate multi‐timepoint consistency, assuming IVIM parameters in normal tissues remain stable during treatment. The image quality is additionally assessed through the noise level in parameter maps, the tissue boundary clarity, and the anatomical structure fidelity.

### 2.8 Statistical analysis

Two sets of statistical analyses were performed. First, for per‐case NMAE comparisons on the AAPM breast IVIM‐dMRI challenge dataset (N = 100 cases), pairwise Wilcoxon signed‐rank tests were used to compare IVIM‐INR against each competing method (least squares, TRR, IVIMNet, ConvNet), with each case treated as one paired observation (each case yielding one NMAE value per parameter per tissue type). Second, for the real clinical data, the ICC was used to quantify multi‐timepoint consistency of IVIM parameters in normal brain tissues (with the clinical target volume (CTV) excluded). Specifically, we adopted the two‐way random‐effects, absolute‐agreement, single‐measurement model, ICC(2,1), following Shrout and Fleiss,^39^ treating voxels as subjects and timepoints as raters. For each patient, each parameter, and each method, a single ICC value was computed across all valid voxels and timepoints within the analysis mask. Pairwise Wilcoxon signed‐rank tests were then applied across patients to compare IVIM‐INR against each competing method. All tests used a two‐sided significance level of p<0.05. The non‐parametric Wilcoxon test was chosen because the underlying NMAE and ICC values may not follow a normal distribution.

## 3 RESULTS

### 3.1 Brain digital phantom evaluation

#### 3.1.1 Performance at different noise levels

**FIGURE 3:**
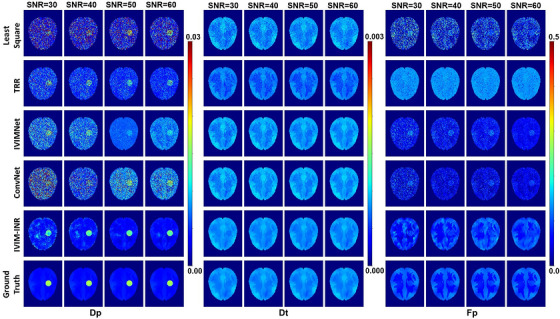
IVIM parameter estimation results for the brain digital phantom at different signal‐to‐noise ratios. From left to right: $D_{p}$, $D_{t}$, and $F_{p}$ parameter maps. IVIM‐INR (second to last row) demonstrates superior denoising effect and parameter estimation accuracy at various noise levels, producing the smoothest parameter distributions closest to the “ground truth” (last row).

Figure 3 visually compares parameter maps from different methods under various SNR conditions. Traditional methods (least squares, TRR) show severe noise contamination at low SNRs and apparent spatial discontinuities even at high SNR conditions. IVIMNet shows slight improvement but is still limited by the voxel‐wise fitting strategy. In contrast, IVIM‐INR produces smooth parameter maps close to the “ground truth” at all SNR levels, effectively balancing noise suppression and detail preservation.

**FIGURE 4:**
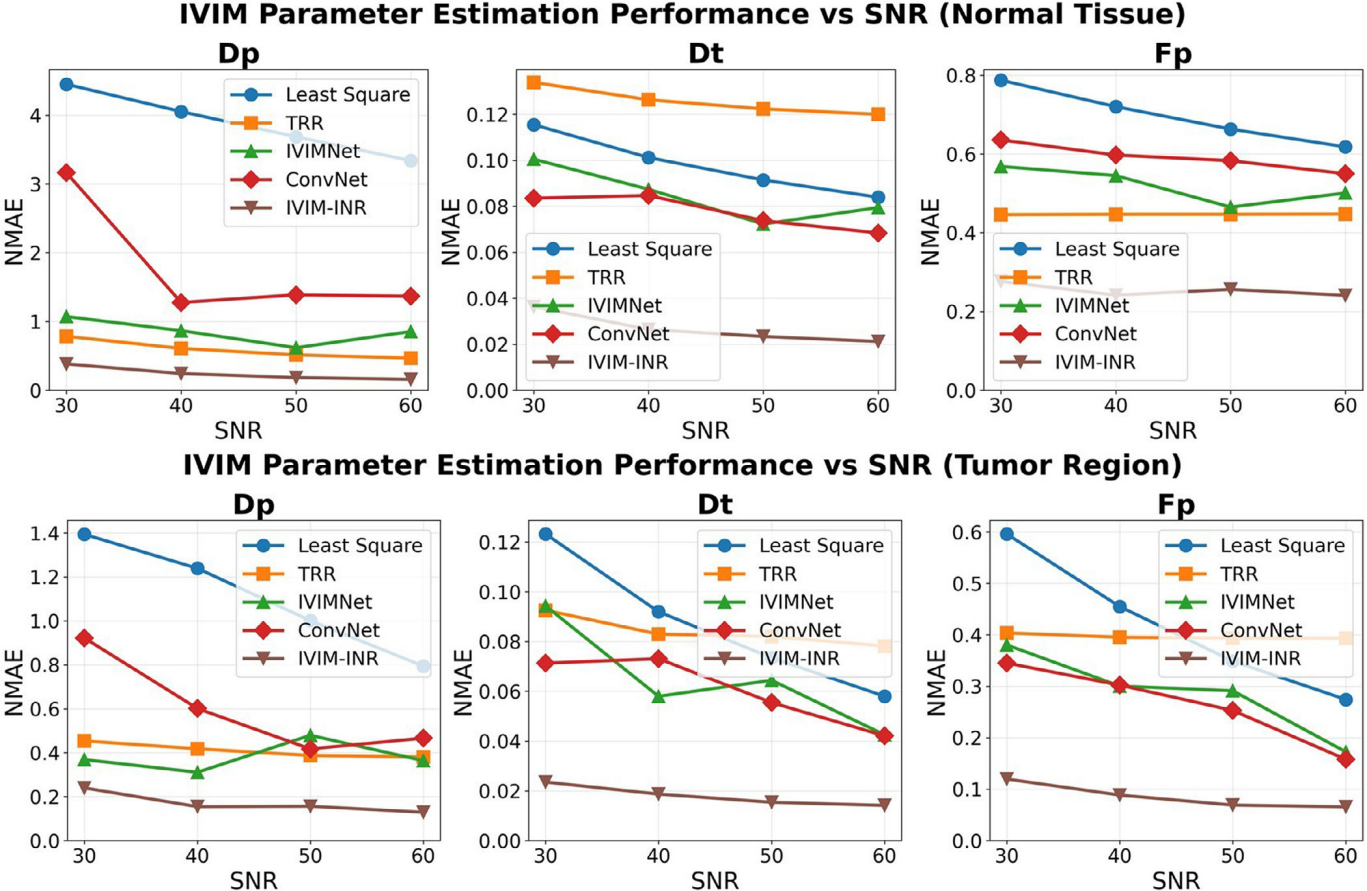
Normalized mean absolute errors at different signal‐to‐noise ratios. The top row shows normal tissues, and the bottom row shows tumor regions. IVIM‐INR exhibits the lowest errors and the most consistent performance improvement trends across all parameters and tissue types.

Quantitative analysis (Figure 4) corroborates the visual observations. In normal tissues, IVIM‐INR's NMAE monotonically decreases with an increasing SNR, with errors for $D_{p}$, $D_{t}$, and $F_{p}$ reducing to 0.16, 0.02, and 0.24, respectively, at SNR = 60. In contrast, traditional methods show high errors and instability. TRR shows limited improvement in fitting performance even with increasing SNR, indicating the existence of a systematic bias. While ConvNet outperforms voxel‐wise methods in $D_{t}$ estimation, its performance in $F_{p}$ and $D_{p}$ is inferior to IVIMNet. For the tumor region, which is much smaller in volume than normal tissue but can be the region of greatest concern in radiotherapy, our IVIM‐INR maintains the best performance, with NMAEs for $D_{p}$, $D_{t}$, and $F_{p}$ at SNR = 50 being 0.16, 0.02, and 0.07, respectively, substantially better than the 0.48, 0.06, and 0.29 of IVIMNet.

#### 3.1.2 Tumor parameter robustness evaluation

**FIGURE 5:**
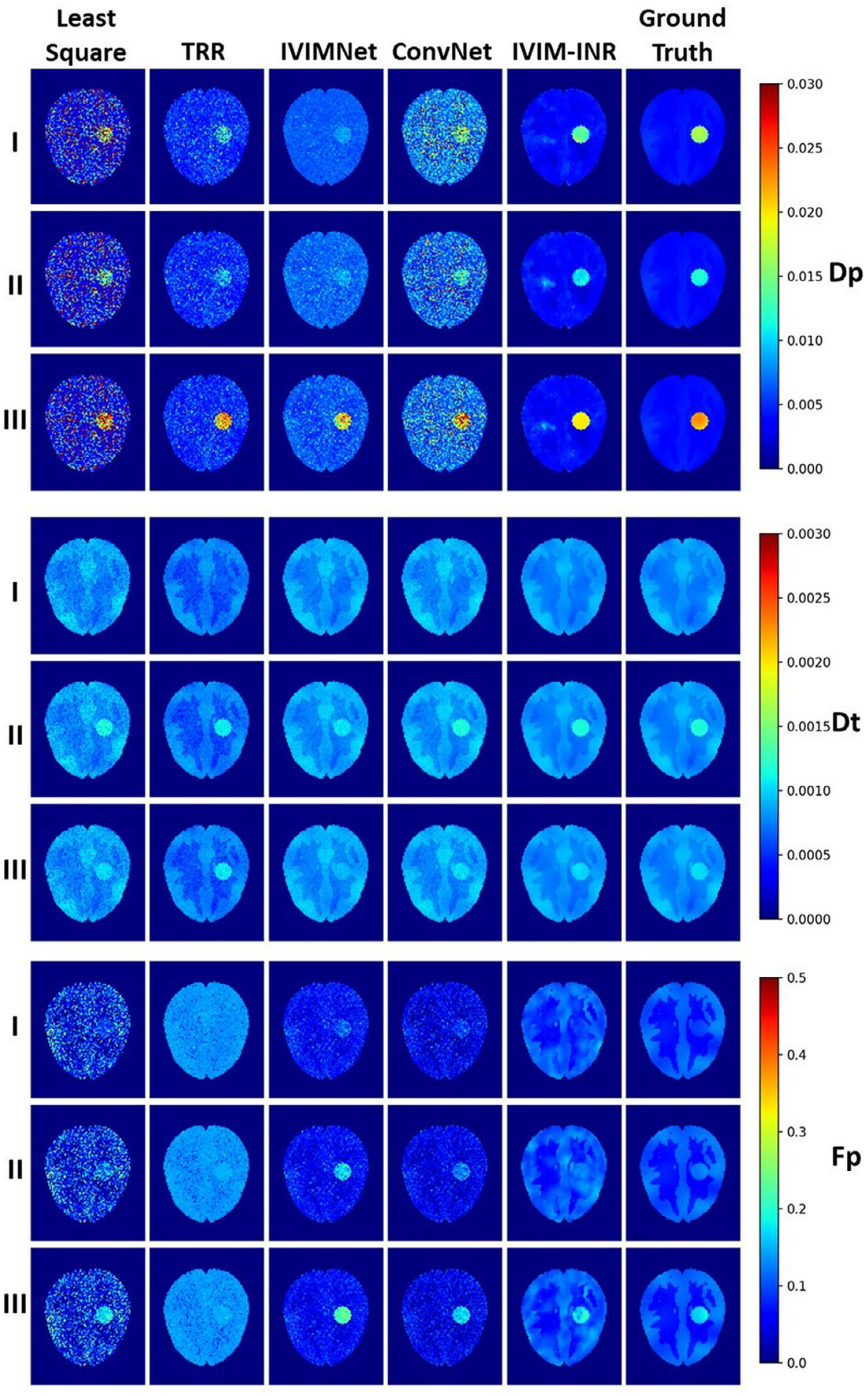
Visualization of IVIM parameter maps for three tumor parameter sets (SNR = 50). Each large block sequentially shows $D_{p}$, $D_{t}$, and $F_{p}$; rows correspond to three tumor parameter sets, and columns correspond to different methods. IVIM‐INR (second to last column) most closely matches the “ground truth” (last column).

**FIGURE 6:**
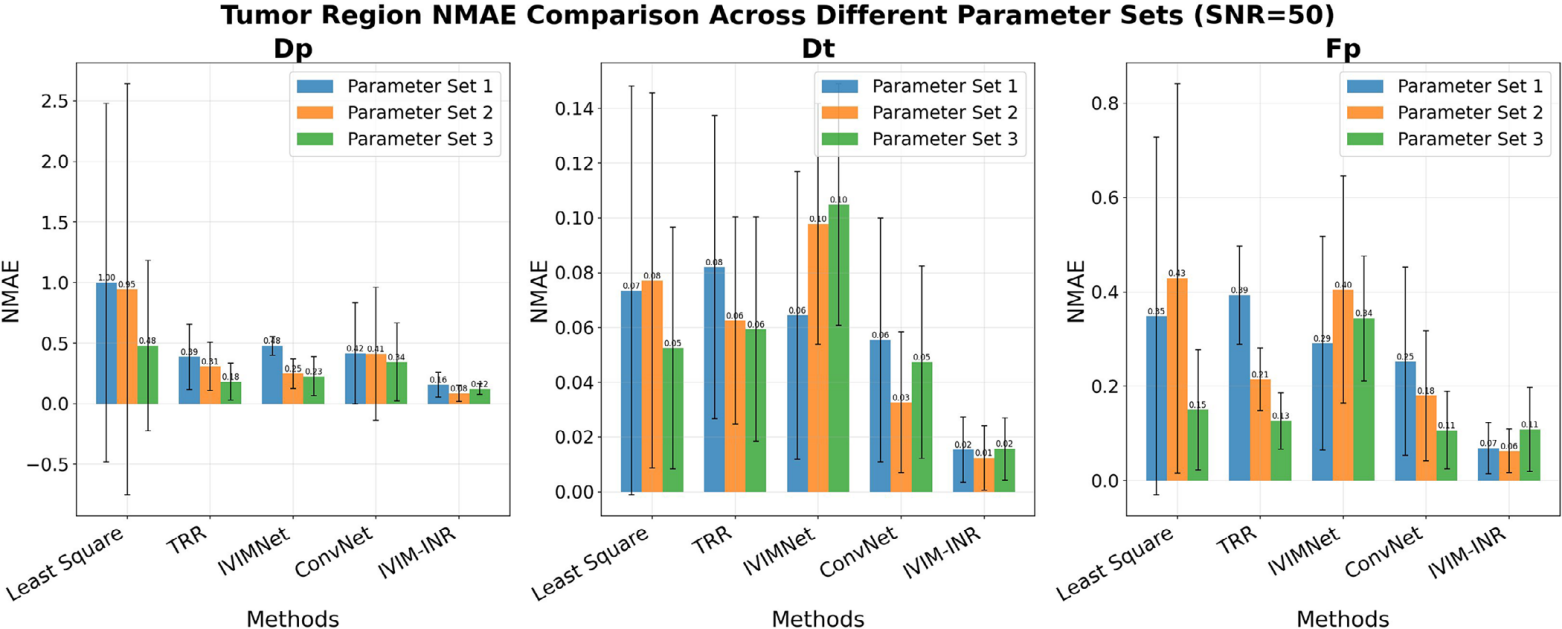
NMAE comparison for three tumor parameter sets. IVIM‐INR demonstrates robustness to parameter variations, while other methods show significant performance fluctuations.

Figures 5 and 6 evaluate different methods' robustness to three different tumor parameter configurations. These three parameter sets are based on representative values from previous literature,^30^ covering the typical variation range of brain tumor IVIM parameters. Visual results (Figure 5) show that all other methods exhibit noise artifacts, blurred tissue boundaries, and loss of contrast between tumor and normal tissue. In contrast, IVIM‐INR most closely matches the “ground truth,” effectively suppressing noise while preserving clear tissue and tumor boundaries. Quantitative evaluation (Figure 6) further confirms IVIM‐INR's superiority. Across three parameter set configurations, IVIM‐INR maintains the lowest NMAE with minimal inter‐group fluctuation, demonstrating robustness to parameter variations. The performance of other methods fluctuates substantially: ConvNet's error in $D_{t}$ estimation for Parameter Set 1 surges to 0.06, nearly doubling that of Set 2; IVIMNet also shows significant instability in $D_{t}$ estimation, with Set 3 error nearly twice that of Set 1. This instability can be particularly challenging in clinical applications, as tumor IVIM parameters are heterogeneous, and models must adapt to different parameter distributions. Overall, IVIM‐INR demonstrates superior robustness across different tumor parameter configurations compared to other methods.

### 3.2 AAPM breast IVIM‐dMRI challenge data evaluation

**FIGURE 7:**
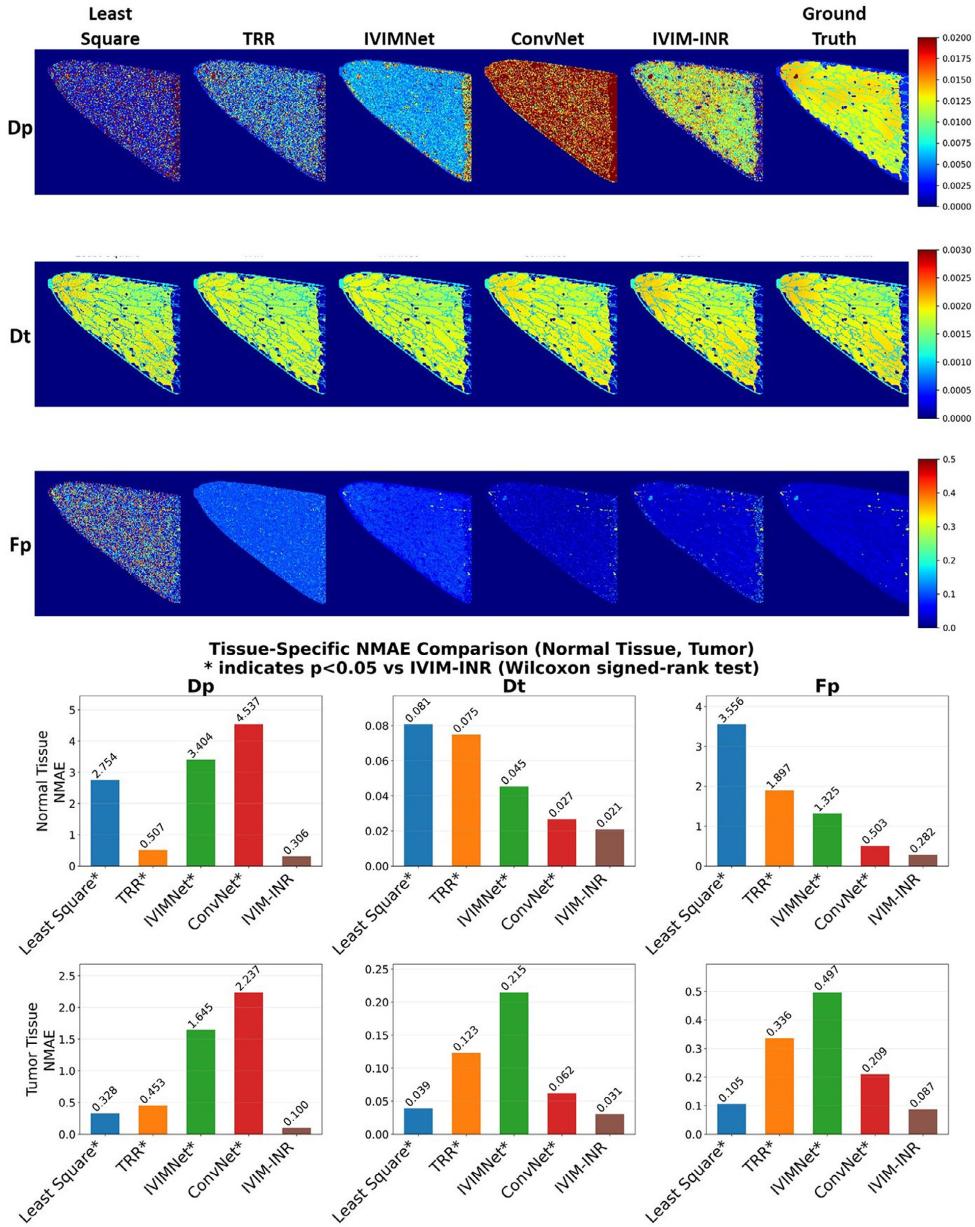
Tissue‐specific evaluation of the VICTRE breast model. The upper panel visualizes the derived parameter maps, and the lower panel shows NMAE statistics for different tissue types. Asterisks indicate statistically significant differences compared to IVIM‐INR (*p* < 0.05).

Figure 7 presents the comprehensive evaluation results on the AAPM challenge data. The upper part shows parameter map visualization comparison across different methods. Pixel‐wise methods (least squares, TRR) are dominated by noise, with parameter maps showing granular artifacts and blurred tissue boundaries. While IVIMNet introduces a network structure, it adopts a similar pixel‐wise fitting strategy and performs poorly at tissue boundaries due to a lack of spatial information integration. ConvNet achieves some degree of spatial smoothing through convolution operations, but excessive smoothing leads to loss of subtle tissue heterogeneity information, and ConvNet's overall $D_{p}$ values are significantly elevated. In contrast, IVIM‐INR produces parameter maps closest to the “ground truth,” suppressing noise while accurately preserving the tissue boundary sharpness and the heterogeneity patterns within lesions.

Quantitative analysis (lower part of Figure 7) further confirms the visual observations. In tumor regions, IVIM‐INR achieves substantial performance improvements: NMAE for $D_{p}$ is only 0.10; NMAE for $D_{t}$ is 0.03, 50% lower than ConvNet (0.06); and NMAE for $F_{p}$ is 0.09. This improvement is consistent across all 100 cases, with the Wilcoxon signed‐rank test confirming statistical significance (*p* < 0.05). Additionally, IVIMNet and ConvNet perform poorly in tumor regions but relatively well in normal tissues, especially for $D_{t}$. This phenomenon may arise because when these methods fit the entire image, all regions contribute to the fitting process. However, since tumor regions in the VICTRE breast model are typically small, they constitute a smaller proportion of training losses compared to normal tissues. Crucially, traditional models lack positional information about where they are fitting, causing tumor parameters to be overshadowed by those of normal tissues, making it difficult for the models to accurately model small tumor regions. In contrast, our model leverages coordinate inputs that inform the model about which region it is fitting, enabling effective differentiation between tissue types and resulting in more accurate fitting for tumor regions.

### 3.3 Real clinical data evaluation

To evaluate method performance in real clinical scenarios, we collected DWI data from seven GBM patients. Since real clinical data lacks “ground‐truth” IVIM parameters, we adopted an evaluation strategy based on temporal consistency. Our core assumption is: during treatment, IVIM parameters in normal brain tissues outside the clinical target volume (CTV) should remain relatively stable; therefore, we can evaluate parameter estimation reliability of different methods by calculating ICC across multiple timepoints. High ICC values indicate that methods produce consistent parameter estimates across different timepoints, reflecting their robustness to noise.^40^

**FIGURE 8:**
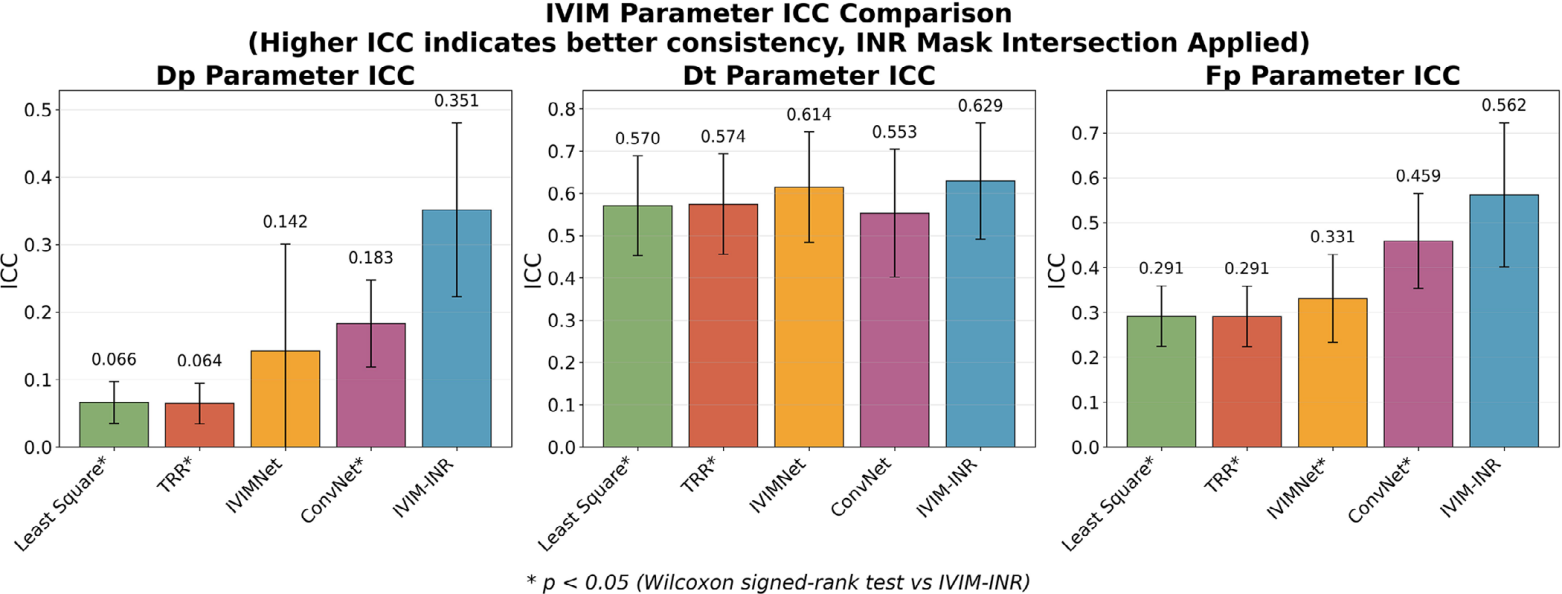
ICC analysis of real GBM data. IVIM‐INR shows the highest temporal consistency, indicating more stable parameter estimation on real clinical data.

**FIGURE 9:**
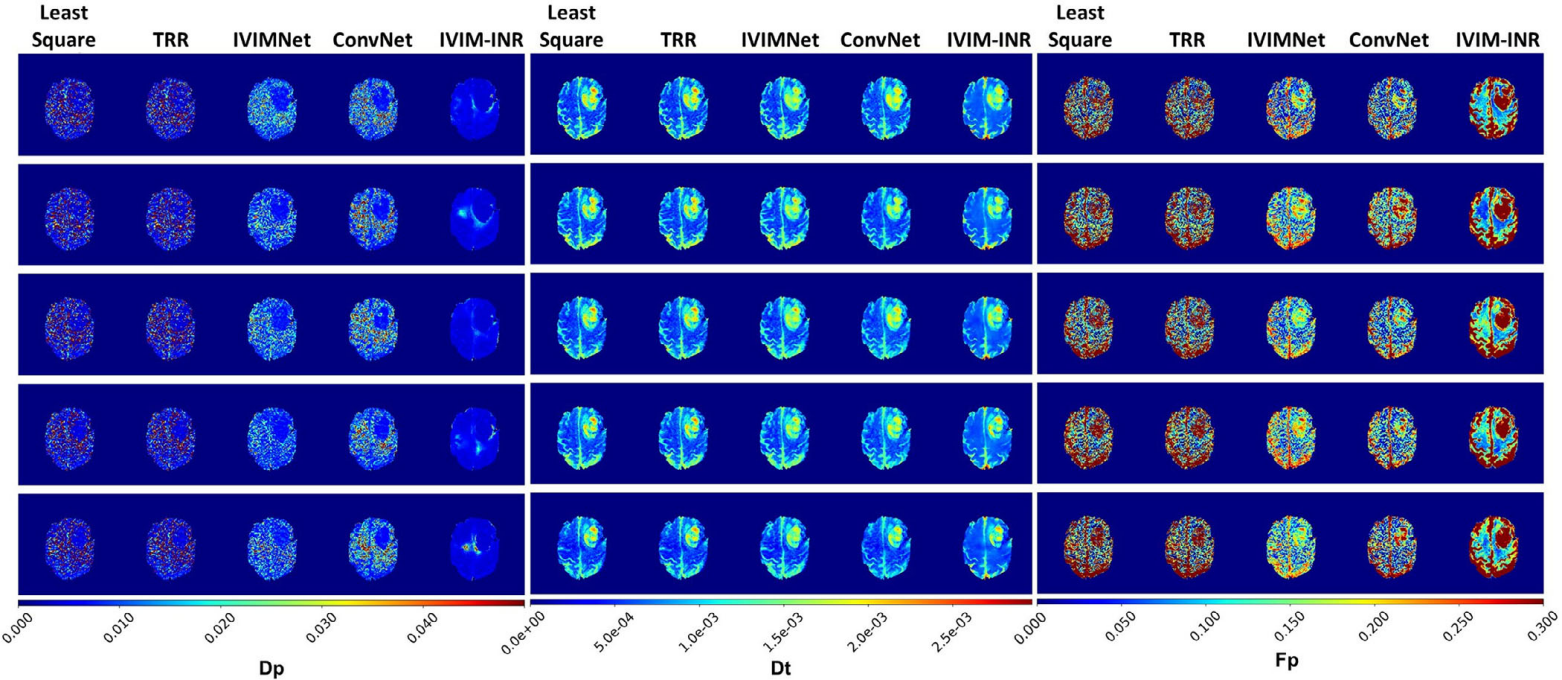
Multi‐timepoint IVIM parameter maps of real patients. Each row shows results from different scan times for the same patient. From left to right, the three panels show $D_{p}$, $D_{t}$, and $F_{p}$ parameter maps, respectively.

Figure 8 shows ICC comparison of different methods for three IVIM parameters in normal brain tissues (excluding the CTV region). IVIM‐INR achieves the highest ICC values for all three parameters. Specifically, for $D_{t}$ estimation, IVIM‐INR's ICC reaches 0.629, significantly higher than least squares (0.570) and TRR (0.574). This advantage is more pronounced for the $F_{p}$ parameter, with IVIM‐INR's ICC at 0.562, while traditional methods are generally below 0.46, with least squares at only 0.291. $D_{p}$ ICC results show a similar pattern, with IVIM‐INR reaching 0.351, while least squares and TRR are both at around 0.06. These results indicate that IVIM‐INR has the highest temporal consistency in normal tissue parameter estimation for real clinical data. Visual comparison (Figure 9) provides intuitive evidence. The figure shows IVIM parameter maps of the same patient at different treatment timepoints. In $D_{p}$ parameter maps, results from least squares and TRR methods are filled with noise grains, making anatomical structures almost unrecognizable; IVIMNet shows improvement but still has apparent noise residuals; in contrast, IVIM‐INR maintains clear anatomical structures at all timepoints with the highest visual consistency between different timepoints. These results demonstrate that IVIM‐INR not only performs excellently on digital phantoms and standardized test data but also maintains the most robust performance when facing the complexity of real clinical data.

### 3.4 Ablation study

**FIGURE 10:**
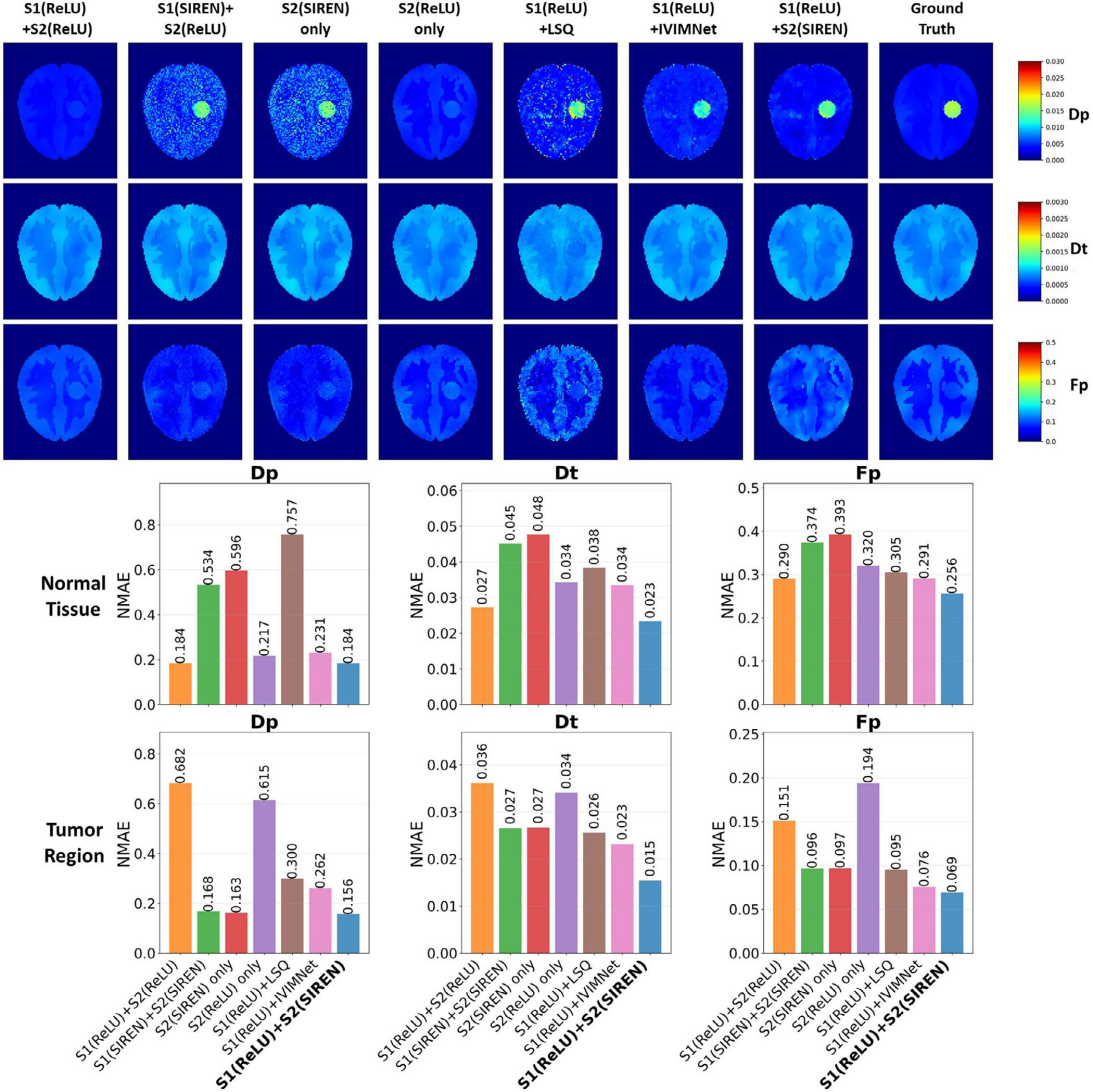
Ablation study of activation function combinations. The upper half visually compares parameter maps, and the lower half shows quantitative NMAE comparisons.

To better demonstrate the rationale of IVIM‐INR's architecture design, we conducted ablation experiments on the brain digital phantom's Parameter Set 1 (SNR = 50), focusing on evaluating the impact of the two‐stage INR design and the activation function selection on the model performance. We compared seven different architecture configurations (Figure 10): (1) S1(ReLU)+S2(ReLU): both stages use ReLU; (2) S1(SIREN)+S2(SIREN): both stages use SIREN; (3) S2(SIREN) only: single‐stage SIREN activation only; (4) S2(ReLU) only: single‐stage ReLU activation only; (5) S1(ReLU)+LSQ: first‐stage INR with ReLU for denoising, followed by traditional least squares for parameter fitting; (6) S1(ReLU)+IVIMNet: first‐stage INR with ReLU for denoising, followed by IVIMNet for parameter fitting; (7) S1(ReLU)+S2(SIREN): our final design.

The comparison results reveal the impact of different architectural choices. The dual‐ReLU configuration achieves good noise suppression but produces over‐smoothed parameter maps with blurred tissue boundaries, particularly losing important heterogeneity information near tumor regions. This is due to ReLU's piecewise linear property, which limits its ability to express high‐frequency details. The dual‐SIREN configuration preserves rich tissue details but amplifies the noise, with parameter maps containing excessive noise grains. It stems from the SIREN's enhanced high‐frequency learning capability, which, however, also amplifies noise. Single‐stage experiments clearly reveal the essential properties of the two activation functions. When using only S2(SIREN), INR directly learns IVIM parameters from noisy data, resulting in noise artifacts with significantly elevated NMAE across all parameters. Using only S2(ReLU) can suppress noise, but it simultaneously suppresses high‐frequency information, leading to detail loss and parameter bias. To validate the necessity of the second‐stage INR architecture, we also tested hybrid approaches combining INR denoising with traditional methods for parameter fitting. The S1(ReLU)+LSQ configuration uses least squares fitting after first‐stage denoising. While it shows improvement over using least squares alone (Figure 4), its performance remains significantly inferior to the complete two‐stage INR design, particularly showing larger errors in $D_{p}$ estimation. The S1(ReLU)+IVIMNet configuration performs better than S1(ReLU)+LSQ, but still underperforms our method, demonstrating that our second‐stage INR is critical to the overall IVIM parameter estimation accuracy.

Quantitative analysis confirms the superiority of the S1(ReLU)+S2(SIREN) combination. In normal tissues, this configuration's NMAEs for $D_{p}$, $D_{t}$, $F_{p}$ are 0.184, 0.023, and 0.256, respectively, lower than all other configurations. In tumor regions, the performance advantage is even more pronounced, with all parameter errors remaining the lowest. Overall, the success of the two‐stage INR design stems from a separate processing strategy for different frequency components. The first stage's ReLU activation function has natural low‐pass filtering properties, effectively suppressing high‐frequency noise while preserving the main signal structure information. Learning intrinsic correlation of signals across multiple *b*‐values, the first stage denoising also maintains fine details through the additional, patch‐based multi‐*b*‐value input. The second stage's SIREN activation function focuses on extracting fine parameter variations from denoised signals, with its periodic activation particularly suitable for capturing high‐frequency details at tissue boundaries and pathological regions.

### 3.5 Parameter sensitivity analysis

**FIGURE 11:**
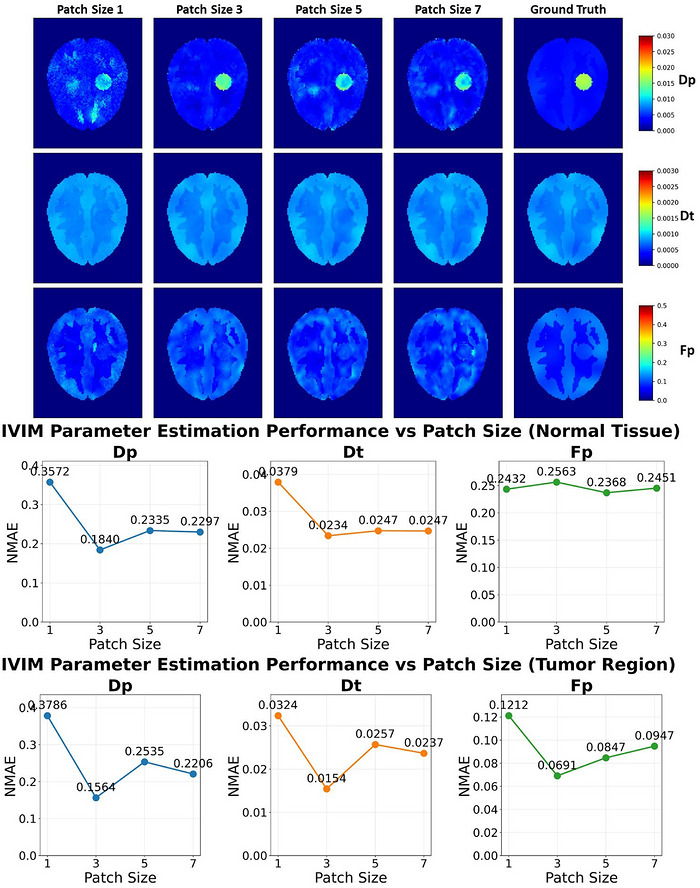
Impact of patch sizes on the parameter estimation accuracy. The top panel visually compares the parameter maps, and the bottom panel shows the NMAE curves in relation to the patch sizes.

Patch size e is a key hyperparameter of IVIM‐INR, determining the amount of local context information the model can access at each spatial position. To systematically evaluate its impact, we test four configurations e∈{1,3,5,7} on the brain digital phantom's Parameter Set 1 (SNR = 50) for consistency with the ablation study. The upper part of Figure 11 shows the impact of different patch sizes on parameter map quality. When e=1, the model can barely access spatial context information, though still retaining some global perception capability through coordinate encoding. Results show that noise is most evident in the e=1 results. e=3 achieves the best balance in visual quality. At this patch size, parameter maps maintain smoothness while preserving tissue structure details. This indicates that 3×3×3 local patches provide sufficient spatial information to guide parameter estimation while avoiding bias from over‐reliance on the local information. When e increases to 5 and 7, while noise suppression is further enhanced, the cost is a loss of fitting accuracy. The lower part of Figure 11 provides more quantitative evidence. In tumor tissues, e=3 achieves the lowest NMAE for all parameters: $D_{p}$ is 0.16, $D_{t}$ is 0.02, and $F_{p}$ is 0.07. Therefore, we chose e=3 as the default setting for all other experiments.

## 4 DISCUSSION

In this study, we proposed IVIM‐INR, a two‐stage framework that introduces implicit neural representations to IVIM parameter estimation, and validated it on brain digital phantoms, the AAPM breast IVIM‐dMRI challenge data, and real GBM clinical data. Across all three settings, IVIM‐INR consistently outperformed traditional voxel‐wise fitting (least squares, TRR), voxel‐wise deep learning (IVIMNet), and the convolutional approach (ConvNet), with particularly pronounced improvements for the perfusion‐related parameters $D_{p}$ and $F_{p}$ that have long been more unstable in IVIM analysis. In the following, we discuss the methodological reasons behind these improvements, their clinical implications, the rationale for key design choices, the practical advantages of the approach, and its current limitations.

A fundamental limitation of traditional voxel‐wise IVIM parameter fitting is the ignorance of spatial continuity in medical images. While least squares and TRR are computationally straightforward, the parameter maps they produce under low SNR conditions are corrupted by amplified noise, limiting clinical value. IVIMNet introduces the nonlinear modeling capability of neural networks but still adopts a voxel‐wise processing paradigm, essentially learning more complex fitting functions without fully utilizing spatial information.^41^ ConvNet introduces spatial processing capability through convolution operations, showing improvement in some scenarios, but its inherent limitations emerge in our experiments: translation invariance assumptions lead to insensitivity to anatomical locations, producing artifacts at tissue boundaries, and local receptive fields limit the understanding of global structures.^16^ In contrast, IVIM‐INR addresses these challenges through INR's continuous function modeling capability. Global spatial perception is a key advantage over convolutional networks: INR naturally obtains perception of the entire imaging volume through coordinate encoding, enabling the network to learn position‐dependent parameter distribution patterns.^17^, ^24^ This is particularly important when dealing with regions of complex anatomical structures and diverse tissue types—for example, in brain imaging, parameter variation patterns at gray‐white matter interfaces differ significantly from periventricular regions, and global position information helps the network adaptively adjust local processing strategies.

The clinical significance of improved IVIM parameter estimation is underscored by the growing body of evidence supporting IVIM's value in tumor diagnosis and treatment monitoring. In glioblastoma, IVIM parameters have demonstrated utility in preoperative grading,^3^, ^42^ survival prediction,^4^, ^43^ and early assessment of treatment response during chemoradiation.^5^ In breast cancer, IVIM has shown promise in differentiating malignant from benign lesions^44^, ^45^ and predicting response to neoadjuvant chemotherapy.^46^ However, the clinical translation of these findings has been hampered by the well‐documented instability of IVIM parameter estimation, particularly for $D_{p}$ and $F_{p}$.^1^, ^2^ Cho et al.^47^ demonstrated that different fitting methods and *b*‐value sampling strategies yield substantially different IVIM parameter estimates in breast cancer, and a meta‐analysis by Liang et al.^45^ attributed significant inter‐study heterogeneity partly to variations in parameter estimation approaches. By providing more stable and accurate parameter maps, especially for the challenging $D_{p}$ and $F_{p}$, IVIM‐INR has the potential to enhance the reliability of IVIM‐based clinical biomarkers and facilitate their translation from research tools to routine clinical practice.

Realizing this clinical potential requires careful attention to several technical choices in our framework, two of which warrant elaboration. The first concerns the additive offset δ in our signal model, which shares the same motivation as fitting $I_{0}$ as a free parameter in IVIMNet^15^ and other IVIM studies.^26^ Both approaches relax the hard constraint that forces the normalized intensity at b=0 to equal exactly 1, allowing the model to accommodate systematic deviations caused by hardware imperfections. We adopt the additive form because it naturally fits within the normalized intensity framework $I\left(b\right)/I_{0}$ used in our formulation, and it allows straightforward regularization that drives δ toward zero to prevent excessive deviation from the standard IVIM model. The second is the Rician bias correction applied during preprocessing, a widely adopted procedure for magnitude DWI data.^28^ Its principal source of uncertainty lies in the estimation of the noise standard deviation σ, since the magnitude of the correction depends directly on this estimate; different σ estimation strategies can yield different correction magnitudes, and more refined approaches such as random‐matrix‐theory‐based estimation have been proposed.^48^ We adopt background‐noise‐based σ estimation because it is a well‐established approach^49^ routinely used in IVIM analysis,^50^ offering mature implementations and good reproducibility. Furthermore, applying the same estimation strategy uniformly across all compared methods ensures that any residual bias from σ estimation does not favor any particular approach.

Beyond accuracy gains, IVIM‐INR offers two practical advantages relevant to clinical translation. First, IVIM‐INR is self‐supervised: the network is trained independently on each individual case using only its own multi‐*b*‐value DWI data, without requiring any external training datasets or ground‐truth labels. This eliminates the need for large annotated training cohorts, inherently avoids the domain shift problem when applied across different scanners, acquisition protocols, or anatomical regions, making IVIM‐INR readily deployable across institutions with diverse imaging setups. Second, the modular two‐stage design provides flexibility in the choice of denoising strategy. The two stages are trained with independent loss functions, so Stage 1 can in principle be replaced by alternative denoising approaches such as conventional spatial filters or random matrix theory‐based methods.^51^ That said, our current Stage 1 employs a ReLU‐based INR that leverages the spectral bias of neural networks and cross‐*b*‐value patch correlations for self‐supervised denoising. The advantage of the current INR‐based denoising is its seamless integration within the unified coordinate‐based framework, its ability to learn case‐specific noise patterns without requiring explicit noise model assumptions, and its integration with spatial features.

Despite these advantages, several limitations should be acknowledged. First, IVIM‐INR adopts a single‐case independent training strategy, which, while ensuring patient‐specific adaptation, fails to leverage population‐level prior knowledge; future work could explore hybrid learning frameworks that combine population statistical priors with individual optimization to improve parameter estimation stability while maintaining specificity.^27^, ^52^ Second, our current implementation is based on the biexponential IVIM model, which, despite being the most widely used, may not fully capture complex diffusion behaviors in regions such as highly perfused organs where tri‐exponential or kurtosis models may be more appropriate; extending the IVIM‐INR framework to accommodate alternative signal models represents a natural future direction. Third, although we validated IVIM‐INR on brain digital phantoms, standardized breast challenge data, and real GBM clinical data, further evaluation across additional anatomical regions (e.g., abdomen, pelvis) and larger patient cohorts would strengthen the evidence for generalizability. Body regions in particular present additional challenges including respiratory and cardiac motion artifacts, greater B1 field inhomogeneity, and more complex tissue compositions with higher perfusion fractions; since IVIM‐INR operates on coordinate‐to‐parameter mappings without region‐specific architectural assumptions, it is in principle applicable to any anatomical site, but the impact of these confounding factors warrants dedicated investigation. Finally, a systematic comparison of different denoising front‐ends within the modular two‐stage architecture could further identify the optimal configuration for various clinical scenarios.

## 5 CONCLUSION

To our knowledge, this study is the first work to apply implicit neural representation to IVIM parameter estimation. Fundamentally different from the traditional paradigm that uses independent fitting for each voxel, our proposed IVIM‐INR framework uses INR to uniformly model the entire IVIM parameter field by taking spatial coordinates as input. This innovative approach changes the way IVIM parameters are estimated by no longer treating each voxel as an isolated optimization problem, but achieving global spatial perception and implicit regularization through continuous function representation. IVIM‐INR provides a novel solution for reliable parameter estimation under low SNR conditions, with the potential to improve IVIM‐based tumor diagnosis, grading, and treatment response assessment. The success of this framework not only demonstrates the great potential of implicit neural representation in medical imaging but also provides new insights for other medical image parameter estimation problems.

## CONFLICT OF INTEREST STATEMENT

The authors declare no conflicts of interest.
